# Keratocyte Differentiation Is Regulated by NF-κB and TGFβ Signaling Crosstalk

**DOI:** 10.3390/ijms231911073

**Published:** 2022-09-21

**Authors:** Xin Zhou, Junhong Li, Ludvig J. Backman, Patrik Danielson

**Affiliations:** 1 Department of Integrative Medical Biology (IMB), Faculty of Medicine, Umeå University, 901 87 Umeå, Sweden; 2 Section of Physiotherapy, Department of Community Medicine and Rehabilitation, Faculty of Medicine, Umeå University, 901 87 Umeå, Sweden; 3 Department of Clinical Sciences, Ophthalmology, Faculty of Medicine, Umeå University, 901 87 Umeå, Sweden

**Keywords:** keratocyte, NF-κB, TGFβ, IL-1, corneal wound healing

## Abstract

Interleukin-1 (IL-1) and transforming growth factor-beta (TGFβ) are important cytokines involved in corneal wound healing. Here, we studied the effect of these cytokines on corneal stromal cell (keratocyte) differentiation. IL-1β treatment resulted in reduced keratocyte phenotype, as evident by morphological changes and decreased expression of keratocyte markers, including keratocan, lumican, ALDH3A1, and CD34. TGFβ1 treatment induced keratocyte differentiation towards the myofibroblast phenotype. This was inhibited by simultaneous treatment with IL-1β, as seen by inhibition of α-SMA expression, morphological changes, and reduced contractibility. We found that the mechanism of crosstalk between IL-1β and TGFβ1 occurred via regulation of the NF-κB signaling pathway, since the IL-1β induced inhibition of TGFβ1 stimulated keratocyte-myofibroblast differentiation was abolished by a specific NF-κB inhibitor, TPCA-1. We further found that Smad7 participated in the downstream signaling. Smad7 expression level was negatively regulated by IL-1β and positively regulated by TGFβ1. TPCA-1 treatment led to an overall upregulation of Smad7 at mRNA and protein level, suggesting that NF-κB signaling downregulates Smad7 expression levels in keratocytes. All in all, we propose that regulation of cell differentiation from keratocyte to fibroblast, and eventually myofibroblast, is closely related to the opposing effects of IL-1β and TGFβ1, and that the mechanism of this is governed by the crosstalk of NF-κB signaling.

## 1. Introduction

Interleukin-1 (IL-1) and transforming growth factor beta (TGFβ) have been proposed to be master regulators of corneal wound healing [1]. IL-1 is known to modulate growth factor production and expression of metalloproteinases and collagenases, and to induce apoptosis of corneal stromal cells (keratocytes) [2,3]. TGFβ is known to stimulate keratocyte–myofibroblast differentiation [4]. In the uninjured cornea, the epithelial basement membrane (EBM) prevents IL-1 and TGFβ from entering the stroma. However, during corneal stromal injury, the EBM is disrupted and both IL-1 and TGFβ are released from epithelial cells and tears into the corneal stroma and act as regulators in the wound healing process. One key event in the repair mechanism is that keratocytes undergo phenotype changes to fibroblasts and myofibroblasts. Although the effects of IL-1 or TGFβ on keratocyte differentiation have been studied [5,6], the interplay of IL-1 and TGFβ in the phenotype changes of keratocytes is unknown.

It is interesting to note that many studies demonstrate opposing effects of IL-1 and TGFβ on stromal cells during corneal injury. For example, IL-1 triggers myofibroblast apoptosis, whereas the presence of TGFβ suppresses the apoptotic induction [4]. Perlecan protein, which is a major component in the EBM, is upregulated by IL-1 but downregulated by TGFβ [7]. IL-1 upregulates the expression of collagenases in corneal stromal cells, whereas TGFβ inhibits collagenase production [8]. While TGFβ is the master regulator of corneal myofibroblast differentiation (reviewed in [1]), it is known that IL-1 inhibits myofibroblast differentiation induced by TGFβ in skin [9,10] and lungs [11].

Stromal wound healing of the cornea requires a stepwise transformation of keratocytes to fibroblasts, and subsequently to myofibroblasts (reviewed in [12]). The transformation of keratocytes to fibroblasts is regulated by growth factors such as TGFβ, FGF-2 and PDGF [6,13]. IL-1, on the other hand, is reported to only stimulate keratocyte proliferation but not fibroblast differentiation based on a lack of morphological changes [6]. However, it has been demonstrated that IL-1 induces a reduction in keratocyte markers after three days of treatment, including keratocan, lumican, aldh3a1, and CD34 [14]. Fibroblast activation and proliferation are important steps in corneal stromal wound healing, which are followed by myofibroblast differentiation and contraction. Consequently, the cell fate of keratocytes after simultaneous exposure to both IL-1 and TGFβ is important to study, considering the potential role of IL-1 in fibroblast activation and proliferation, as well as the opposing effects of TGFβ. 

TGFβ has three isoforms (β1, β2, and β3) identified in mammals. TGFβ1 plays a dominant role among the three in wound repair processes [15]. There are two isoforms of IL-1 presented in the eye: IL-1α and IL-1β. The IL-1β concentration is transiently elevated during corneal wound healing [16,17], while the IL-1α level is not changed in response to wounding in mice [17]. Considering their relevance to corneal wound healing, IL-1β and TGFβ1 were chosen for this study.

The potential mechanism of crosstalk between IL-1 and TGFβ could involve the NF-κB signaling pathway. IL-1 is known to induce activation of this pathway [18,19,20]. NF-κB activation is in turn involved in the activation of Smad7, which is a key regulator of TGFβ signaling by negative feedback loops [21]. Smad7 both degrades activated TGFβ receptors and interacts with transcriptional repressors in the nuclei [22,23]. Whether there is IL-1 and TGFβ crosstalk via NF-κB induced Smad7 in corneal stromal cells remains unknown.

In light of all this, we hypothesize that IL-1 and TGFβ crosstalk determines the cell fate of keratocytes. The purpose of this study is to delineate the phenotype changes of keratocytes in response to IL-1 and TGFβ treatment, and the underlying mechanisms, with focus on the NF-κB and TGFβ signaling pathways.

## 2. Results

### 2.1. IL-1β Induces Loss of Keratocyte Properties Independent of TGFβ1

**Figure 1:**
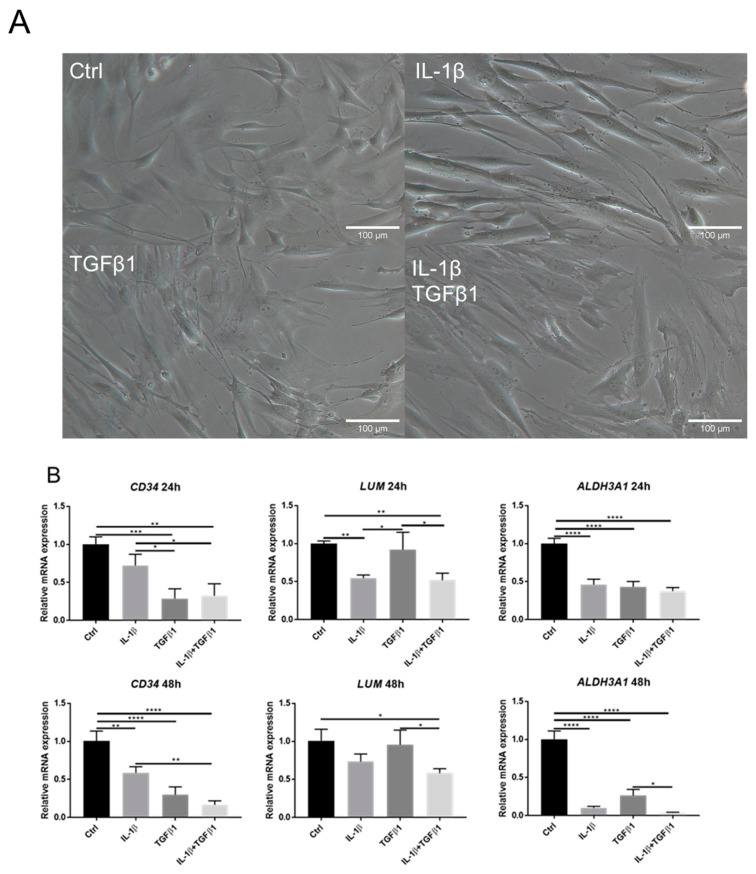
IL-1β and TGFβ1 treatment resulted in morphological changes and reduced expression of keratocyte markers. (**A**) Representative images demonstrating morphological changes in keratocytes exposed to IL-1β (1 ng/mL) and/or TGFβ1 (1 ng/mL) for 24 h. (**B**) Gene expression levels of lumican (LUM), aldehyde dehydrogenase 3 family member A1 (ALDH3A1), and CD34 after treatment with 1 ng/mL IL-1β and/or 1 ng/mL TGFβ1, assessed by qPCR at 24 h and 48 h (*n* = 3). Data are presented as mean ± standard deviation. * *p* < 0.05, ** *p* < 0.01, *** *p* < 0.001, **** *p* < 0.0001.

1 ng/mL IL-1β was used to treat keratocytes with/without simultaneous treatment of 1 ng/mL TGFβ1 to study the effect of IL-1 and TGFβ on the phenotype changes of keratocytes. Distinct morphological changes were observed by the stimulation of TGFβ1. As shown in Figure 1A, cells exposed to TGFβ1 exhibited large and spread-out morphology, i.e., a myofibroblast-like appearance. Live cell imaging videos also showed morphological changes over time in response to IL-1β and/or TGFβ1 treatments (Supplemental Files).

As shown in Figure 1B, reduced expression of keratocyte markers including keratocan (KERA), lumican (LUM), and aldehyde dehydrogenase 3 family member A1 (ALDH3A1), was observed following treatment with IL-1β as compared to the control after 24 h. TGFβ1 induced reduction in CD34 and ALDH3A1, as compared to the control, after 24 h. Simultaneous addition of TGFβ1 and IL-1β induced a reduction in the three examined keratocyte markers (CD34, ALDH3A1, and LUM), as compared to the control, after 24 h. IL-1β resulted in reduced expression of LUM, as compared with after the TGFβ1 treatment. The opposite was seen for CD34 after 24 h. Similar trends between all groups were observed after 48 h. 

### 2.2. IL-1β Inhibits TGFβ1 Mediated Myofibroblast Differentiation

**Figure 2:**
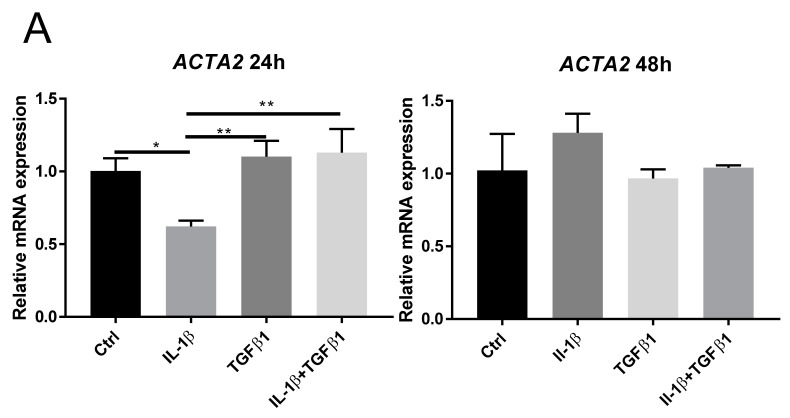

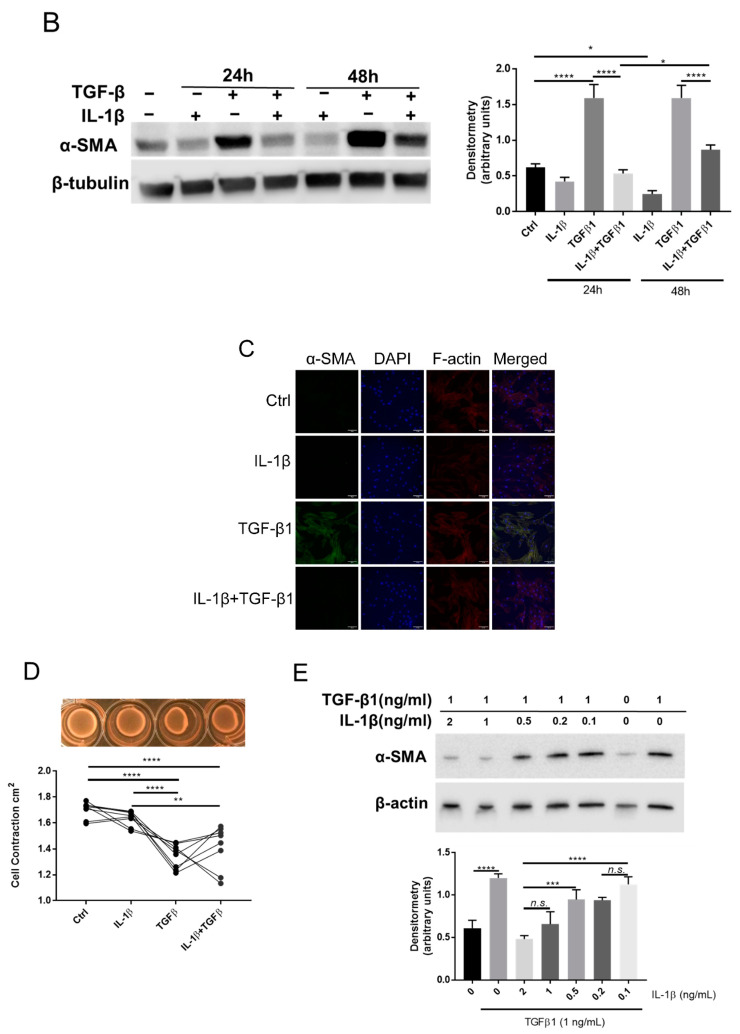
IL-1β suppressed TGFβ1-induced myofibroblast differentiation. (**A**) mRNA expression of ACTA2 in cells treated with 1 ng/mL TGFβ1 and/or 1 ng/ ml IL-1β after 24 h and 48 h (*n* = 3). (**B**) Western blot analysis showed the effect of IL-1β (1 ng/mL) and/or TGFβ1 (1 ng/mL) on α-SMA expression in keratocytes. Tubulin served as a loading control. (**C**) Representative α-SMA (green) and F-actin (red) immunostaining of keratocytes treated with IL-1β (1 ng/mL) and/or TGFβ1 (1 ng/mL) in 10% FBS for 24 h. (**D**) Gel contraction assay showing the effect of IL-1β (1 ng/mL) and/or TGFβ1 (1 ng/mL) on the contractibility of corneal stromal cells. Representative images of contracted gels after different treatment are shown in the upper panel. Quantitative analysis was shown in the lower panel. The lines indicate contraction of the same primary corneal keratocyte cell-line after different treatments (*n* = 8). (**E**) α-SMA expression was measured by Western blot. Various doses of IL-1β were added in the presence of TGFβ1 (1 ng/mL) for 24 h. Data are presented as mean ± standard deviation. * *p* < 0.05, ** *p* < 0.01, *** *p* < 0.001, **** *p* < 0.0001. *n.s.* (not significant).

The morphological changes seen after treatment (Figure 1A) prompted us to study the effect of IL-1β and/or TGFβ1 on corneal stromal cell differentiation. ACTA2 is the gene coding for alpha-smooth muscle actin (α-SMA). IL-1β alone reduced ACTA2 mRNA expression as compared to all other groups at 24 h. No significant differences in ACTA2 mRNA expression were observed at 48 h (Figure 2A). Western blot showed that α-SMA expression markedly increased in response to TGFβ1 treatment. However, simultaneous administration of IL-1β and TGFβ1 resulted in reduced α-SMA expression, as compared to TGFβ1 alone, at 24 h and 48 h (Figure 2B). Immunocytochemistry of α-SMA confirmed that TGFβ1 alone induced positive immunoreactions, and that only weak reactions were detected in cells treated with TGFβ1 and IL-1β simultaneously (Figure 2C). TGFβ1 induced increased contraction, measured by gel contraction assay, as compared to control and IL-1β alone, after 48 h. No significant changes in contraction were noticed when TGFβ1 and IL-1β stimulation was given simultaneously (Figure 2D). To test whether the inhibitory effect of IL-1β on TGFβ1 induced α-SMA expression is dose-dependent, different doses of IL-1β were used simultaneously as cells were stimulated by 1 ng/mL TGFβ1. As shown in Figure 2E, lower doses of IL-1β (0.5 ng/mL and below) were unable to inhibit TGFβ1 induced α-SMA expression, suggesting that the inhibition of α-SMA induction by IL-1 β is dose-dependent.

### 2.3. Opposing Effects of IL-1β and TGFβ1 in NF-κB and TGFβ Signaling

**Figure 3:**
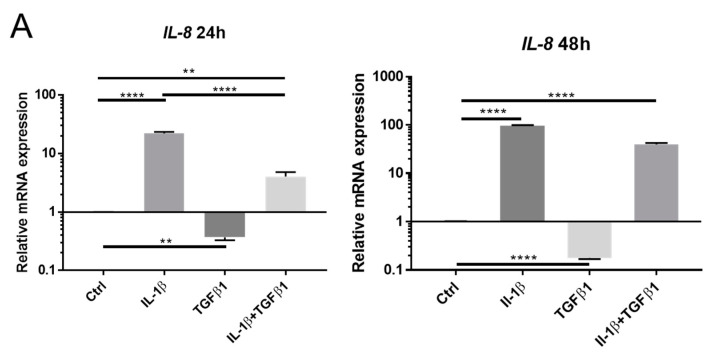

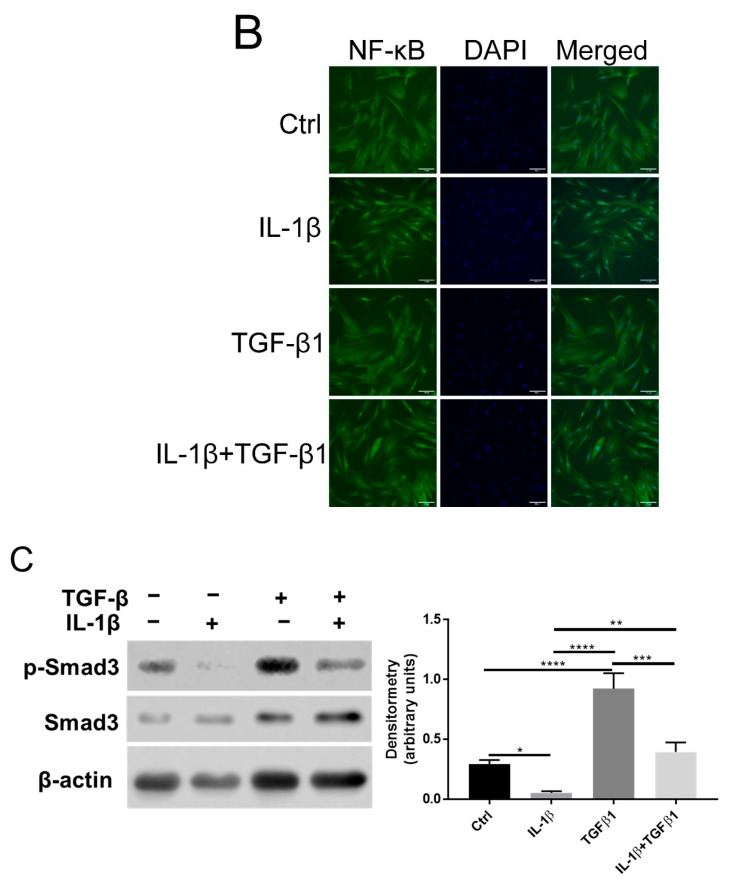
IL-1β activation of NF-κB signaling. (**A**) mRNA expression of IL-8 in cells treated with IL-1β (1 ng/mL) and/or TGFβ1 (1 ng/mL) after 24 h and 48 h (*n* = 3). (**B**) Representative images of NF-κB (red) localization in stromal cells treated with IL-1β (1 ng/mL) and/or TGFβ1 (1 ng/mL) for 24 h. (**C**) Western blot analysis showing the effect of IL-1β (1 ng/mL) and/or TGFβ1 (1 ng/mL) on Smad3 phosphorylation in keratocytes after 24 h. Actin served as a loading control. Densitometry is normalized to β-actin. Data are presented as mean ± standard deviation. * *p* < 0.05, ** *p* < 0.01, *** *p* < 0.001, **** *p* < 0.0001.

It is well known that IL-1 stimulates NF-κB signaling via binding of IL-1 to its preferred receptor. Our previous studies have shown that IL-8 is a reliable surrogate to reveal NF-κB activation in keratocytes [24,25]. In the present study, we found that IL-1β stimulation resulted in increased IL-8 mRNA expression, as compared to the control, at 24 h and 48 h. Contrarily, TGFβ1 downregulated the basal level of IL-8, as compared to the control, at 24 h and 48 h. Simultaneous stimulation of IL-1β and TGFβ1 resulted in increased expression of IL-8, as compared to the control or TGFβ1 alone, at both 24 h and 48 h (Figure 3A). IL-1β facilitated NF-κB nucleus translocation, as revealed by immunostaining. The accumulation of NF-κB in the nucleus stimulated by IL-1β was attenuated with the presence of TGFβ1 (Figure 3B). Together, these results confirm that NF-κB signaling is activated by IL-1β, whereas TGFβ1 attenuates the NF-κB signaling. IL-1β stimulation resulted in reduced expression of Smad3 phosphorylation, as compared to control, at 24 h. TGFβ1 induced an increase in the expression of Smad3 phosphorylation, and this increase was reduced by simultaneous addition of IL-1β at 24 h (Figure 3C). These results suggest that NF-κB signaling interferes with TGFβ signaling, as Smad3 phosphorylation is a common marker of TGFβ signaling activation.

### 2.4. Myofibroblast Differentiation Can Be Regulated by a Selective NF-κB Signaling Inhibitor

**Figure 4:**
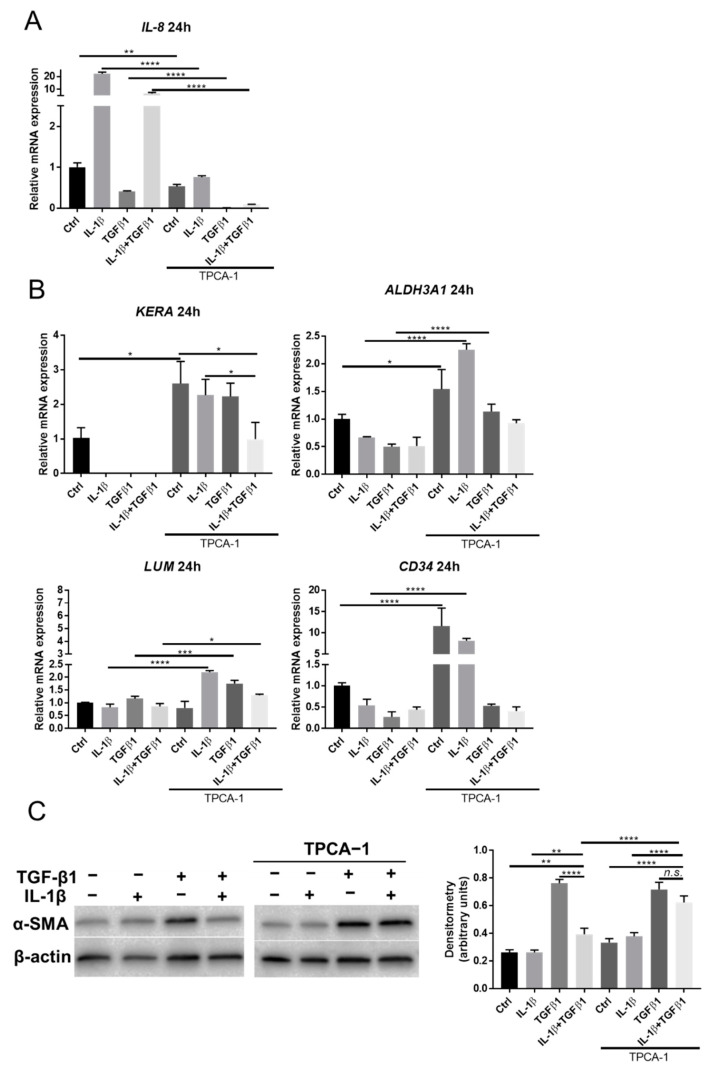
TPCA-1 reversed the effects of IL-1β on corneal stromal cells. (**A**) mRNA expression of IL-8 in cells stimulated with IL-1β (1 ng/mL) and/or TGFβ1 (1 ng/mL) with/without 1 μM TPCA-1 after 24 h (*n* = 3). (**B**) mRNA expression of keratocyte markers in cells treated with IL-1β (1 ng/mL) and/or TGFβ1 (1 ng/mL) with/without 1 μM TPCA-1 (*n* = 3). (**C**) Western blot analysis showing the effect of IL-1β (1 ng/mL) and/or TGFβ1 (1 ng/mL) on α-SMA expression in keratocytes with/without 1 μM TPCA-1 after 24 h. Actin served as a loading control. KERA: Keratocan. Data are presented as mean ± standard deviation. * *p* < 0.05, ** *p* < 0.01, *** *p* < 0.001, **** *p* < 0.0001. *n.s.* (not significant).

Based on our previous findings, we know that administration of a selective NF-κB inhibitor, TPCA-1, reduces the expression of IL-8 [25] and that the keratocyte phenotype is preserved under IL-1β stimulation [14]. In the present study, we showed that administration of 1 μM TPCA-1 reduces IL-8 expression in all groups, as compared to cells without TPCA-1 stimulation (Figure 4A). Expression of keratocyte markers KERA, ALDH3A1, LUM, and CD 34 was generally increased following stimulation of TPCA-1, as compared to cells without TPCA-1 stimulation at 24 h (Figure 4B). Furthermore, TPCA-1 stimulation abolished the inhibitory effect of IL-1β on TGFβ1-induced α-SMA expression, as revealed by Western blot analysis (Figure 4C). In summary, TPCA-1 effectively inhibits NF-κB activation, as measured by IL-8 expression, and reverses the effects of IL-1β on keratocytes, as measured by keratocyte marker expression (qPCR) and α-SMA expression (Western blot).

### 2.5. Smad7 Is Involved in the Crosstalk between NF-κB and TGFβ Signaling

**Figure 5:**
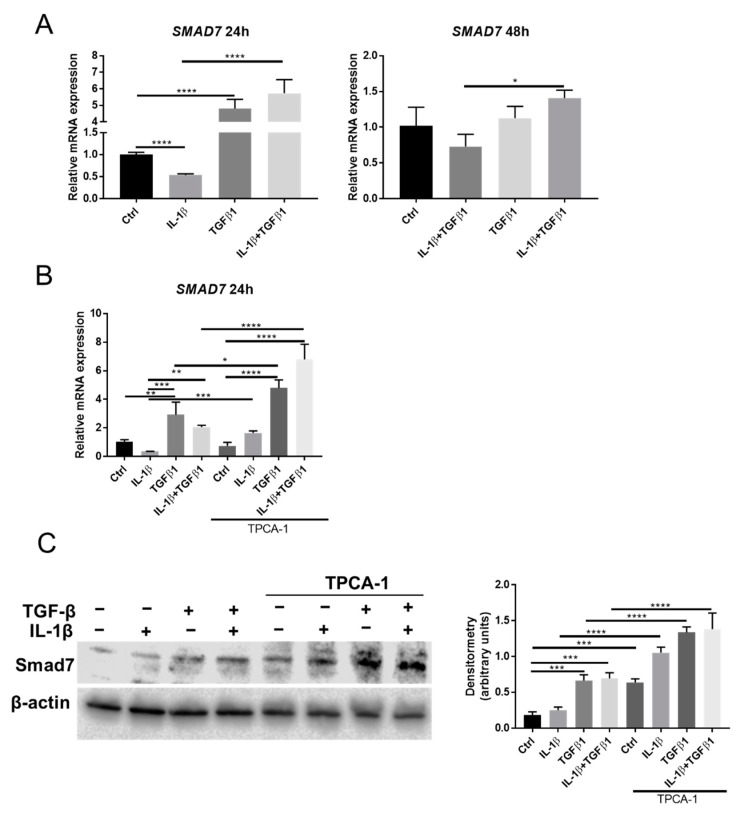
Smad7 expression is regulated by NF-κB and TGFβ1 signaling. (**A**) mRNA expression of Smad7 in cells treated with IL-1β (1 ng/mL) and/or TGFβ1 (1 ng/mL) after 24 h or 48 h (*n* = 3). (**B**) mRNA expression of Smad7 expression in cells treated with IL-1β (1 ng/mL) and/or TGFβ1 (1 ng/mL) with/without 1 μM TPCA-1 after 24 h (*n* = 3). (**C**) Western blot analysis showing the effect of IL-1β (1 ng/mL) and/or TGFβ1 (1 ng/mL) on Smad7 expression in cells with/without 1 μM TPCA-1 after 24 h. Actin served as a loading control. Data are presented as mean ± standard deviation. * *p* < 0.05, ** *p* < 0.01, *** *p* < 0.001, **** *p* < 0.0001.

Smad7 is a key factor to link the NF-κB and TGFβ signaling pathways [21,26]. We found that Smad7 expression is reduced after exposure to IL-1β and that TGFβ1 upregulates Smad7 expression after 24 h, as evidenced by RT qPCR and Western blot (Figure 5A,C). Inhibition of NF-κB signaling, by TPCA-1, resulted in increased Smad7 mRNA expression in all groups except in the control (Figure 5B). The protein expression of Smad7 was elevated in all groups when TPCA-1 was included (Figure 5C). Collectively, the data suggest that NF-κB signaling is involved in suppressing the expression of Smad7. These results contradict the hypothesis that IL-1β could inhibit TGFβ signaling via induction of Smad7, which means that alternative pathways are involved in the IL-1β inhibition of TGFβ signaling by NF-κB activation.

## 3. Discussion

TGFβ and IL-1 both enter the stroma at high levels in the absence of EBM shortly after stromal injury [27,28]. These two cytokines are suggested to be the master regulators of corneal wound healing (reviewed in [1]). Although many aspects of their roles in corneal wound healing have been well studied, their combined effects on keratocyte differentiation and the underlying signaling crosstalk are not documented. In this study, we provide a suggested explanation of the functional role of IL-1β and TGFβ1 in keratocyte cell differentiation. Both IL-1β and TGFβ1 activate keratocytes to enter a fibroblast stage. However, the ratio of IL-1 and TGFβ1 concentration determines the differentiation fate from fibroblast to myofibroblast. TGFβ1 induces myofibroblast differentiation, which is blocked by IL-1β via NF-κB signaling. Smad7 plays an important role in the crosstalk between TGFβ and NF-κB signaling.

Previous studies have reported that IL-1 only confers mitogenic activity [6,13], i.e., is not involved in cellular differentiation [6]. However, they used 10 ng/mL recombinant human IL-1α to treat rabbit keratocytes [6]. In our study, we used 1 ng/mL recombinant human IL-1β to treat keratocytes derived from human donors. Our results show that IL-1β facilitates keratocyte–fibroblast transformation, as confirmed by morphological changes and reduced expression of keratocyte markers. In addition, IL-1β reduced the TGFβ induced α-SMA expression, thus preserving cells in a fibroblastic stage. These results emphasize the important function of IL-1β during the early phase of wound healing, as fibroblast activation and proliferation are essential for corneal stromal healing. Notably, there are high basal levels of α-SMA (Figure 2E) and p-SMAD3 (Figure 3C) in keratocytes before administration of TGFβ1, suggesting partial activation of fibroblasts. The 2% fetal bovine serum (FBS) used here contains many growth factors, including TGFβ, which might have stimulated TGFβ1 signaling, to some degree, in the control. Note that addition of IL-1β resulted in reduced p-SMAD3 expression compared to the untreated control (Figure 3C), suggesting that IL-1β suppressed the background level of TGF-β/SMAD signaling in cells cultured with 2% FBS. The effect of FBS was also evident by the addition of TPCA-1, which increased the expression level of keratocyte markers above the control. Overall, these data suggest that FBS components affect the background level of NF-κB and TGFβ signaling. These data are in line with our central findings, supporting that NF-kB signaling counteracts TGFβ signaling and induces loss of keratocyte markers. Since IL-1 is known to activate the NF-κB signaling pathway, we used a selective inhibitor, TPCA-1. By using TPCA-1, the TGFβ1-induced α-SMA expression was preserved despite the presence of IL-1β, which otherwise reduced the TGFβ1induced α-SMA expression. This result proves that NF-κB activation interferes with TGFβ1 signaling. In addition, administration of TPCA-1 resulted in higher expression of keratocyte markers in all TPCA-1-treated groups, suggesting that NF-κB signaling also plays an important role in the expression of keratocyte markers. This is consistent with our previous results which showed that inhibition of NF-κB signaling, either by TPCA-1 or siRNA, results in increased expression of keratocyte markers [14]. Our results suggest that IL-1β has a dual role in corneal stromal cell differentiation, as it both induces fibroblast activation and inhibits myofibroblast differentiation. NF-κB signaling plays a central role in both of these aspects. Further studies are needed to decipher the molecular mechanisms involved in the regulation of cell fate by NF-κB signaling.

Many studies have demonstrated that NF-κB and TGFβ signaling pathways converge at common target genes. For example, TGFβ can synergize with IL-1 to enhance the expression of collagen type VII in human dermal fibroblasts [29]. TGFβ can activate NF-κB via both Smad dependent [30,31,32] and independent [26,33,34] pathways. However, TGFβ can also inhibit NF-κB signaling by increasing IκB transcription via Smad7 [35]. Vice versa, NF-κB can inhibit TGFβ signaling by inducing Smad7 expression [21,26]. Yee-Yung et al. proposed that the TGFβ signaling activation can induce the expression of inhibitory Smad7 via a negative feedback loop, and that Smad7 may increase IkBα to inhibit NF-κB activation and inflammatory response [36]. This proposed signaling mechanism is consistent with our findings. We showed that Smad7 expression is upregulated by TGFβ1, and the fact that Smad7 suppresses NF-κB-driven inflammatory response by activating the NF-κB inhibitor IκBα could explain the reduction in IL-8 expression for keratocytes treated with TGFβ1. These findings also help to explain the reduction in Smad7 after IL-1β treatment. We suggest that IL-1β induced NF-κB activation directly inhibits the TGFβ signaling pathway, and that Smad7 is also downregulated in response to suppressed TGFβ signaling. This is supported by the fact that IL-1β decreased Smad7 expression, and that the addition of TPCA-1 significantly increased Smad7 expression in IL-1β treated cells. Our data suggest a signal axis of TGFβ–Smad7–NF-κB. However, the way by which NF-κB activation downregulates TGFβ signaling, remains elusive. Studies have shown that NF-κB signaling induces Smad7 to inhibit TGFβ signaling [21,26]. However, this signal axis might not apply to keratocytes, as we found that NF-κB signaling, induced by IL-1β, reduces Smad7 expression, and furthermore that inhibiting NF-κB signaling increases Smad7 expression. Thus, the inhibition of α-SMA expression by IL-1β stimulation is therefore regulated by other signaling pathways. Further studies are needed to unravel alternative pathways that link NF-κB signaling activation and TGFβ signaling inhibition.

It is interesting to note that the effect of IL-1β and/or TGFβ1 on α-SMA (Figure 2) and SMAD7 expression (Figure 5) observed at 24 h decreased at 48 h. We speculate that this phenomenon is explained by a negative feedback signaling in response to IL-1β and/or TGFβ1 stimulation after 24 h. The increase in SMAD7 expression at 24 h after TGFβ1 stimulation supports our suggested explanation, since SMAD7 serves as a negative regulator of TGFβ-induced SMAD signaling. The IL-1 signaling pathway is conserved for its transient nature. For example, IL-1 receptor (IL-1R) is degraded after activation of the IL-1 signaling [37,38,39]. Several negative-feedback pathways can also be triggered by IL-1 to switch off IL-1R signaling [40,41,42]. Our results suggest a dynamic response of keratocytes to cytokine stimulation, which might be related to the timing of keratocyte differentiation during wound healing.

**Figure 6:**
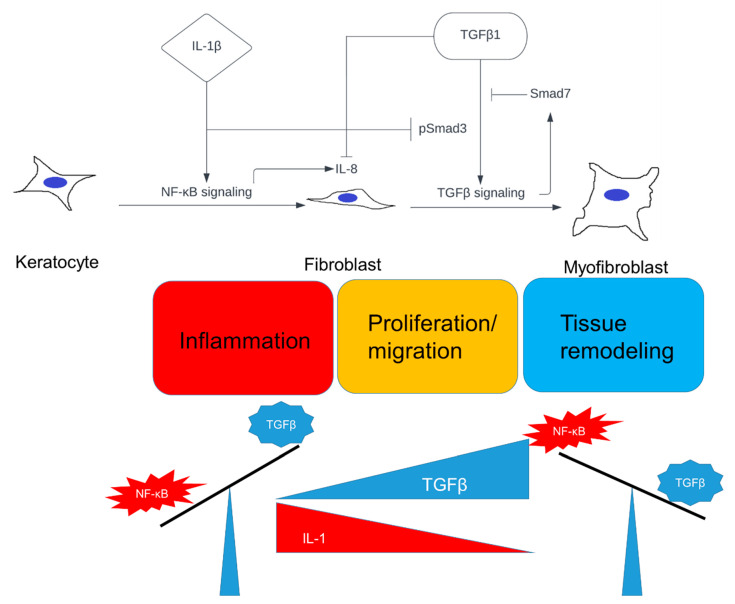
Proposed model for the role of IL-1 and TGFβ in corneal stromal cell differentiation during wound healing. A high concentration of IL-1 is released from injured corneal epithelial cells and immune cells and stimulates transition of keratocytes into corneal fibroblast, as well as proliferation and migration during the early phase of stromal injury. IL-1-induced NF-κB signaling serves as the dominant signaling pathway. Myofibroblast differentiation is prevented due to the inhibitory effect of NF-κB signaling on TGFβ-induced signaling. However, TGFβ from epithelial cells, tears, and possibly stromal cells, accumulates over time during wound healing. Thus, the balance eventually shifts to TGFβ dominant signaling, which leads to cell-cycle arrest and myofibroblast differentiation. The wound healing stage enters the wound closure phase with the maturation of contractile myofibroblast.

In conclusion, we provide evidence that IL-1β and TGFβ1 determine cell fate of corneal stromal cells (keratocytes), which might play an important role in corneal wound healing (Figure 6). Keratocytes are firstly transformed into fibroblasts, and are eventually differentiated into myofibroblasts, which corresponds to the phases of wound healing [43,44,45,46,47,48]. We propose that in the initial stages of corneal stromal wound healing, the concentration of IL-1 is increased due to its release from injured corneal epithelial cells, and possibly from immune cells, which in turn leads to fibroblast activation, proliferation, and migration, predominantly driven by NF-κB signaling. The local concentration of TGFβ accumulates during the progress of wound healing due to its continuous release from epithelial cells and tears, and possibly from stromal cells as well. Stromal cells enter a myofibroblast stage when the ratio of IL-1/TGFβ is reversed. The whole process is governed by the opposing effects between NF-κB and TGFβ signaling, and their balance determines cell fate. Further studies are needed to reveal the mechanism of how NF-κB signaling inhibits TGFβ signaling in corneal stromal cells in vitro. The hypothesis furthermore remains to be tested in vivo, including the study of how the IL-1/TGFβ ratio within the corneal stroma changes over time during wound healing.

In our study, we demonstrated that two cytokines, IL-1β and TGFβ1, play opposite roles in regulating keratocyte differentiation, which underline the crosstalk between NF-κB and TGFβ signaling. We hypothesize, that a timely orchestrated ratio of the two cytokines might coordinate the corneal wound healing phases. Adjusting the concentration of IL-1 and/or TGFβ, or fine-tuning the NF-κB and/or TGFβ signaling, to allow keratocytes to enter the appropriate healing stage at the appropriate time, might be an approach to achieve more optimal corneal wound healing in the future.

## 4. Materials and Methods

### 4.1. Isolation and Culture of Human Keratocytes

Isolation and culture of primary keratocytes were performed as previously described [49]. Healthy human corneal tissue was received for research purposes from the Tissue Establishment, Eye Bank Umeå, at the University Hospital of Umeå, Sweden. The tissue originated from deceased individuals who had chosen, when alive, to donate their corneas postmortem for transplantation and research, according to Swedish law. Grafts not used for transplantation, or the leftover tissue from healthy grafts used for transplant surgery, were delivered to the laboratory for research purposes. The Regional Ethical Review Board in Umeå reviewed the protocol and determined it to be exempt from the requirement for approval (2010-373-31M). The study followed the principles of the Declaration of Helsinki.

Cell isolation and culture were performed as described previously [24]. Corneal samples were scraped using a sterile scalpel to remove any remaining epithelial or endothelial cells, before being washed in sterile Hanks’ balanced salt solution (Invitrogen, Carlsbad, CA, USA). The remaining stromal layer was cut into 1–2 mm^2^ pieces with a scalpel and then digested with 1 mg mL^−1^ collagenase (Sigma, St. Louis, MO, USA) overnight at 37 °C. The suspension was centrifuged and the pellet was cultured in DMEM/F-12 media (Gibco, Carlsbad, CA, USA) supplemented with 2% fetal bovine serum (FBS; Gibco, Carlsbad, CA, USA) and placed in a humidified incubator at 37 °C with 5% CO_2_. Media was changed every third day until the cells reached confluence. Confluent cells were detached with 0.05% Trypsin-EDTA and split into a 1:3 ratio. Cells were passaged until passage 2 and then frozen and stored in liquid nitrogen. In this study, cells from different donors were used for each experiment. Cells from passages 3 to 7 were used for experiments.

### 4.2. Reagents for Experiments

Recombinant IL-1β (1 ng/mL) (R&D Systems, Minneapolis, MN, USA) and/or TGFβ1 (1 ng/mL) (R&D Systems, Minneapolis, MN, USA) were added to the culture medium with or without TPCA-1 (1 μM) (Selleck Chemicals, Houston, TX, USA) and cultured at 37 °C with 5% CO_2_ for 1–2 days. Cells were pre-treated with TPCA-1 for 1 h before IL-1β and/or TGFβ1 addition. The concentration of TGFβ1 to induce myofibroblast differentiation is based on literature [4,50]. The concentrations of IL-1β and TPCA-1 used were adapted from our previous study [14].

### 4.3. RNA Extraction and qRT-PCR

This process involved 0.25 × 10^6^ keratocytes being seeded into 6-well plates in DMEM/F-12 medium supplemented with 2% FBS. At the end of the experiment, cells were washed with PBS and frozen at −80 °C before extraction (three experimental replicates/group). Extraction of mRNA was performed using the RNA extraction kit (Qiagen, Venlo, Netherlands, # 74106) according to the manufacturer’s instructions. Subsequently, high-capacity cDNA reverse transcription kit (Thermo Fisher, Waltham, MA, USA) was used to reverse transcribe RNA into cDNA. To determine the gene expression, TaqMan Gene Expression Assays (Applied Biosystems, Carlsbad, CA, USA) were used. cDNA was run using ViiA7 Real-Time PCR system and analyzed with its software (Applied Biosystems, Carlsbad, CA, USA). Gene expression was measured by TaqMan Gene Expression Assay (Applied Biosystems, Carlsbad, CA, USA) and calculated by 2^−∆∆Ct^ method. All probes used for real-time PCR (Applied Biosystems, Carlsbad, CA, USA) are summarized in Table 1. β-actin was used as the reference gene for normalization.

### 4.4. Western Blot

Western blot analysis involved 0.25 × 10^6^ keratocytes being seeded into 6-well plates in DMEM/F-12 medium supplemented with 2% FBS. At the end of the experiment, cells were washed with PBS and frozen at −80 °C before extraction (three experimental replicates/group). Cells were freeze-thawed and further lysed in RIPA (radioimmunoprecipitation) lysis buffer (Thermo Fisher, Waltham, MA, USA) supplemented with protease and phosphatase inhibitor cocktail (Sigma, St. Louis, MO, USA, #P1860). Total protein concentration was determined with the BCA assay (Thermo Fisher, Waltham, MA, USA). Samples containing 20 µg of protein were separated on SDS-polyacrylamide gels and transferred to PVDF membranes (Thermo Fisher, Waltham, MA, USA). Membranes were blocked in 5% bovine serum albumin in TBS-T for 1 h before staining with primary antibodies overnight at 4 °C. After washing, the membranes were stained with HRP-conjugated secondary antibodies for 1 h at room temperature before incubation with ECL solution and then analyzed in an Odyssey Fc Dual-Mode Imaging System (LI-COR Biotechnology, Nebraska, USA). Densitometry was performed using Image J analysis software (NIH). β-actin was used to normalize target protein expression. Densitometry analysis was calculated as follows: Intensity of the protein of interest was divided by the intensity of β-actin. All antibodies used are summarized in Table 2.

### 4.5. Immunocytochemistry

This process involved 0.25 × 10^6^ keratocytes being seeded into 6-well plates in DMEM/F-12 medium supplemented with 2% FBS (three experimental replicates/group). At the end of the experiment the cultured cells were washed three times with PBS and fixed with 10% formalin for 10 min, and then transferred into PBS at 4 °C. The samples were permeabilized with 1% Triton X-100 and blocked with 1:20 diluted normal serum. F-actin was stained by phalloidin (Thermo Fisher, Waltham, MA, USA, #A22287) and α-SMA was stained by Alexa Fluor 488 conjugated antibody (1:400) (Thermo Fisher, Waltham, MA, USA, # 53976082). For NF-κB p65 staining, cells were incubated with the NF-κB p65 antibody (1:200) for 60 min at room temperature. After washing, cells were incubated with secondary IgG conjugated with Alexa Fluor 555 at 1:300 concentration for 60 min. DAPI (Thermo Fisher, Waltham, MA, USA, #62248) was used to stain the nuclei of the cells. Subsequently, cells were washed three times and the images were acquired by Leica Thunder Widefield Microscope (Leica AG, Heerbrugg, Switzerland). Negative staining control, by omitting primary antibody, resulted in no unspecific signals for NF-κB p65 staining. All antibodies used are summarized in Table 2.

### 4.6. Contraction Assay

Bovine collagen solution type I PureCol^®^ (Advanced BioMatrix, Carlsbad, CA, USA, #5074-35ML) was diluted in DMEM/F-12 Medium to get a final collagen gel concentration of 5 mg/mL (0.5%). Five hundred microliter of the collagen gel was mixed with 0.25 × 10^6^ keratocytes supplemented with 0.1% FBS in a 24well plate format. Collagen gels were allowed to polymerize for 1 h at 37 °C. The gels were then released from the walls of the plate using a pipette tip. Next, collagen gels were covered with DMEM/F-12 0.1% FBS supplemented with 1 ng/mL TGFβ1 and/or 1 ng/ ml IL-1β in the experimental group. Collagen gels only containing cells and 0.1% FBS served as control. Pictures were taken 2 days after gels were released (eight experimental replicates/group). Image J software (NIH, Bethesda, MD, USA) was used to measure the area of the gels. Representative images of contraction assays are shown.

### 4.7. Live-Cell Imaging

Cellular morphology was assessed in real time using the Incucyte^®^ S3 Live-Cell Analysis System (Sartorius, Ann Arbor, MI, USA). Cells were seeded in 6-well plates in DMEM/F-12 medium supplemented with 2% FBS (three experimental replicates/group). Thereafter, the cells were placed in the Incucyte^®^ System and treated with IL-1β (1 ng/mL) and/or TGFβ1 (1 ng/mL) for 48 h. The software was adjusted to obtain 9 images per well every 1 h over the 48 h period of treatments.

### 4.8. Statistics

Data were analyzed using GraphPad Prism 7 (GraphPad Software, San Diego, CA, USA) software. One-way analysis of variance (ANOVA) with Tukey’s multiple comparison (post hoc) test was performed in comparisons between more than two groups. Differences were considered statistically significant at a *p*-value of  <0.05. All experiments were repeated successfully at least three times (i.e., at least three separate experiments were performed with cells isolated from different donors). All experimental samples were prepared in triplicates (*n* = 3). Gel contraction assay was performed on 8 samples per group (*n* = 8).

## Figures and Tables

**Table 1:** Probes used for qPCR from Applied Biosystems.

Gene Name	Gene Symbol	Assay ID
Keratocan	*KERA*	Hs00559941_m1
Lumican	*LUM*	Hs00929860_m1
Aldehyde dehydrogenase 3 family member A1	*ALDH3A1*	Hs00964880_m1
CD34	*CD34*	Hs00990732_m1
Alpha 2 smooth muscle actin	*ACTA2*	Hs00909449_m1
*β*-actin	*ACTB*	4352667

**Table 2:** Antibodies used for immunostaining and western blot.

Antibody	Company	Code	Applications
α-SMA	Abcam	ab5694	WB
NF-κB p65	Cell Signaling	8242	IF
Smad7	R&D systems	MAB2029	WB
Smad3 (phospho S423 + S425)	Abcam	ab52903	WB
Smad2/3	Cell Signaling	3102	WB
*β*-actin	Cell Signaling	4967	WB
*β*-tubulin	Abcam	Ab6046	WB
Anti-rabbit Alexa Fluor 555	Thermo Fisher	A32794	IF
Anti-rabbit IgG HRP-linked	Cell Signaling	7074	WB
Anti-mouse IgG HRP-linked	Cell Signaling	7076	WB

## Data Availability

Any data or material that support the findings of this study can be made available by the corresponding author upon request.

## Supplementary Materials

The following supporting information can be downloaded at: https://www.mdpi.com/article/10.3390/ijms231911073/s1.
